# Portable dynamic ultrasonography is a useful tool for the evaluation of suspected syndesmotic instability: a cadaveric study

**DOI:** 10.1007/s00167-022-07058-4

**Published:** 2022-07-26

**Authors:** N. C. Hagemeijer, B. Lubberts, J. Saengsin, R. Bhimani, G. Sato, G. R. Waryasz, G. M. M. J. Kerkhoffs, C. W. DiGiovanni, D. Guss

**Affiliations:** 1 Foot and Ankle Research and Innovation Laboratory, Massachusetts General Hospital, Harvard Medical School, Boston, USA; 2 Department of Orthopaedic Surgery and Sports Medicine, Amsterdam UMC location University of Amsterdam, Meibergdreef 9, Zuidoost, 1105 AZ Amsterdam, The Netherlands; 3 Department of Orthopaedic Surgery, Faculty of Medicine, Chiang Mai University, 110 Inthawarorot, Sri Phum subdistrict, Mueang Chiang Mai District, Chiang Mai 50200, Chiang Mai, Thailand; 4 Foot and Ankle Service, Department of Orthopaedic Surgery, Massachusetts General Hospital, Yawkey Building, 55 Fruit St, Boston, MA 02114 USA; 5 Newton-Wellesley Hospital, Harvard Medical School, Massachusetts General Hospital, 2014 Washington St, Newton, MA 02462 USA; 6 Academic Center for Evidence Based Sports Medicine (ACES), Academic Medical Centre Amsterdam, Meibergdreef 9, 1105 AZ Amsterdam, the Netherlands; 7 Amsterdam Collaboration for Health and Safety in Sports (ACHSS), IOC Research Center, Amsterdam, the Netherlands; 8 Department of Orthopaedic Surgery, Massachusetts General Hospital, 55 Fruit Street, Boston, MA 02114 USA

**Keywords:** Ankle, Ligaments, Imaging, Diagnostic ultrasound, Syndesmosis, Tibiofibular joint

## Abstract

**Purpose:**

Portable ultrasonography (P-US) is increasingly used to diagnose syndesmotic instability. The aim of this study was to evaluate syndesmotic instability by measuring the distal tibiofibular clear space (TFCS) in a cadaveric model using P-US with progressive stages of syndesmotic ligamentous transection under external rotation stress.

**Methods:**

Ten fresh lower leg cadaveric specimens amputated above the proximal tibiofibular joint were used. Using P-US, the TFCS was evaluated in the intact stage and after progressive sectioning of the (1) anterior–inferior tibiofibular ligament (AITFL), (2) interosseous ligament (IOL), and (3) posterior–inferior tibiofibular ligament (PITFL). The TFCS was measured in both the unstressed (0 Nm) state and with 4.5, 6.0, 7.5, and 9.0 Nm of external rotation stress using a bone hook placed on the first metatarsal bone at each stage of ligamentous transection stage using both P-US and fluoroscopy.

**Results:**

When assessed with P-US, partial syndesmotic injury encompassing the AITFL and IOL resulted in significant TFCS widening at 4.5 Nm of external rotation torque when compared to intact state with a TFCS-opening of 2.6 ± 2 mm, *p* = 0.01. In contrast, no significant differences in TFCS were detected using fluoroscopy. Only a moderate correlation was found between P-US and fluoroscopy.

**Conclusion:**

P-US is a useful tool in diagnosing syndesmotic instability during external rotation stress examination. TFCS-opening increased as additional ligaments of the syndesmosis were transected, and application of 4.5 Nm torque was sufficient to detect a difference of 2.6 mm after the IOL cut.

## Introduction

Ankle sprains are among the most commonly reported sports injuries, and up to 18% of ankle sprains involve the syndesmotic ligament complex [1, 20, 22, 41]. Colloquially referred to as a high ankle sprain, the most critical aspect of initial assessment is distinguishing stable from unstable injuries. High ankle sprains are especially caused by a forced external rotation injury, which may result in sequential injury to the anterior inferior tibiofibular ligament (AITFL), interosseous ligament (IOL), and posterior inferior tibiofibular ligament (PITFL) [14, 24, 37].

Failure to diagnose syndesmotic instability can lead to longstanding and often permanent patient morbidity [11, 19, 33, 35]. On the other hand, the diagnosis of subtle syndesmotic instability remains challenging. Stressed radiographs have low sensitivity, and MRI readily detects injury, but does not allow for dynamic joint evaluation [23].

Arthroscopy has traditionally served as the gold standard; however, it remains a costly and invasive procedure that, furthermore, does not afford a contralateral comparison [17].

Weight-bearing computed tomography (WBCT) is a promising technique to distinguish stable from unstable injuries by virtue of allowing a bilateral, 3-dimensional (3D) assessment of the distal syndesmosis under physiologic load [2, 6, 7, 16]. It may not be readily available in many clinical settings, and the axial stress applied may not entirely replicate the rotational or sagittal stress that can be applied manually to detect more subtle instability.

Dynamic portable ultrasound (P-US) is increasingly used because of its ready availability, low cost, and ability to dynamically assess the syndesmosis while affording a contralateral comparison. It is a promising and reliable technique to evaluate the tibiofibular clear space (TFCS) under external rotation stress and sagittal fibular translation, as shown in previous studies [1, 18, 30, 31].

The aim of this study was to evaluate the use of P-US to diagnose syndesmotic instability in a cadaveric model by measuring the TFCS under external rotation stress during progressive stages of ligamentous transection, and compare these results with fluoroscopic measurements. It was hypothesised that P-US could differentiate the intact from the sequent transection stages and that findings are correlated to those detected using fluoroscopy.

## Materials and methods

### Specimens

The use of cadaveric tissue models for biomechanical testing was exempt by the IRB 2016P001295/MGH. Ten fresh–frozen, nonpaired lower leg cadaveric specimens amputated above the proximal tibiofibular joint were used in this study (mean age at the time of death, 64 years; range, 29–91 of which 7 were male). Before starting the experiment, a fluoroscopic (OrthoScan FD Pulse C-Arm, OrthoScan, Scottsdale, Arizona) and arthroscopic (Arthrex, Naples, Florida) evaluation was performed. Specimens were excluded if there were any signs of ankle osteoarthritis or previous trauma. Soft tissues were maintained to simulate in vivo conditions. Specimens were thawed at room temperature and secured to a board using four 4-mm Schanz-type pins inserted anteroposteriorly into the tibia.

### Sequential transection of ligaments

Each specimen underwent evaluation with P-US and fluoroscopy in the intact state and thereafter at each stage of sequential ligamentous transection including the, (1) AITFL, (2) distal 10 cm of the IOL, and (3) PITFL. For the ligament transection, an open incision was made. The ligament transection was performed sharply with a surgical blade No. 10 by a specialised foot and ankle orthopaedic surgeon (JS).

### Experimental setup

**Fig. 1:**
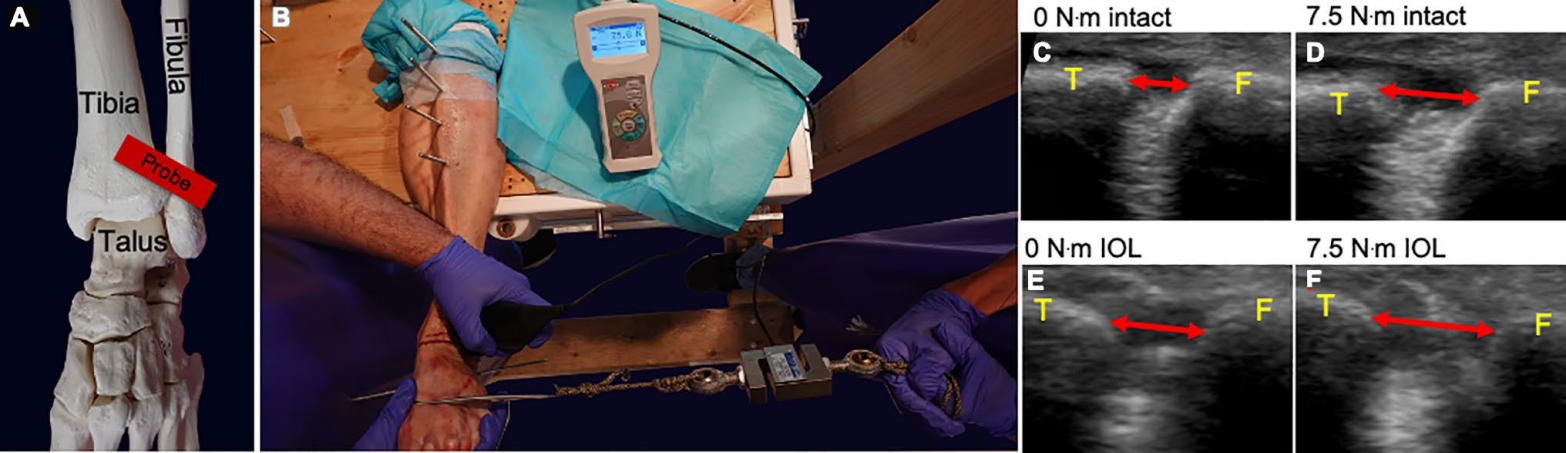
P-US experimental setup. **A** Bone model of probe position. **B** Ultrasound experimental set up. **C–F** Ultrasound image of the TFCS at 0 and 7.5 Nm, at the intact stage (**C**, **D**) and after AITFL + IOL transection (**E**, **F**). Red arrows resemble the TFCS measurements. *T* tibia, *F* fibula

**Fig. 2:**
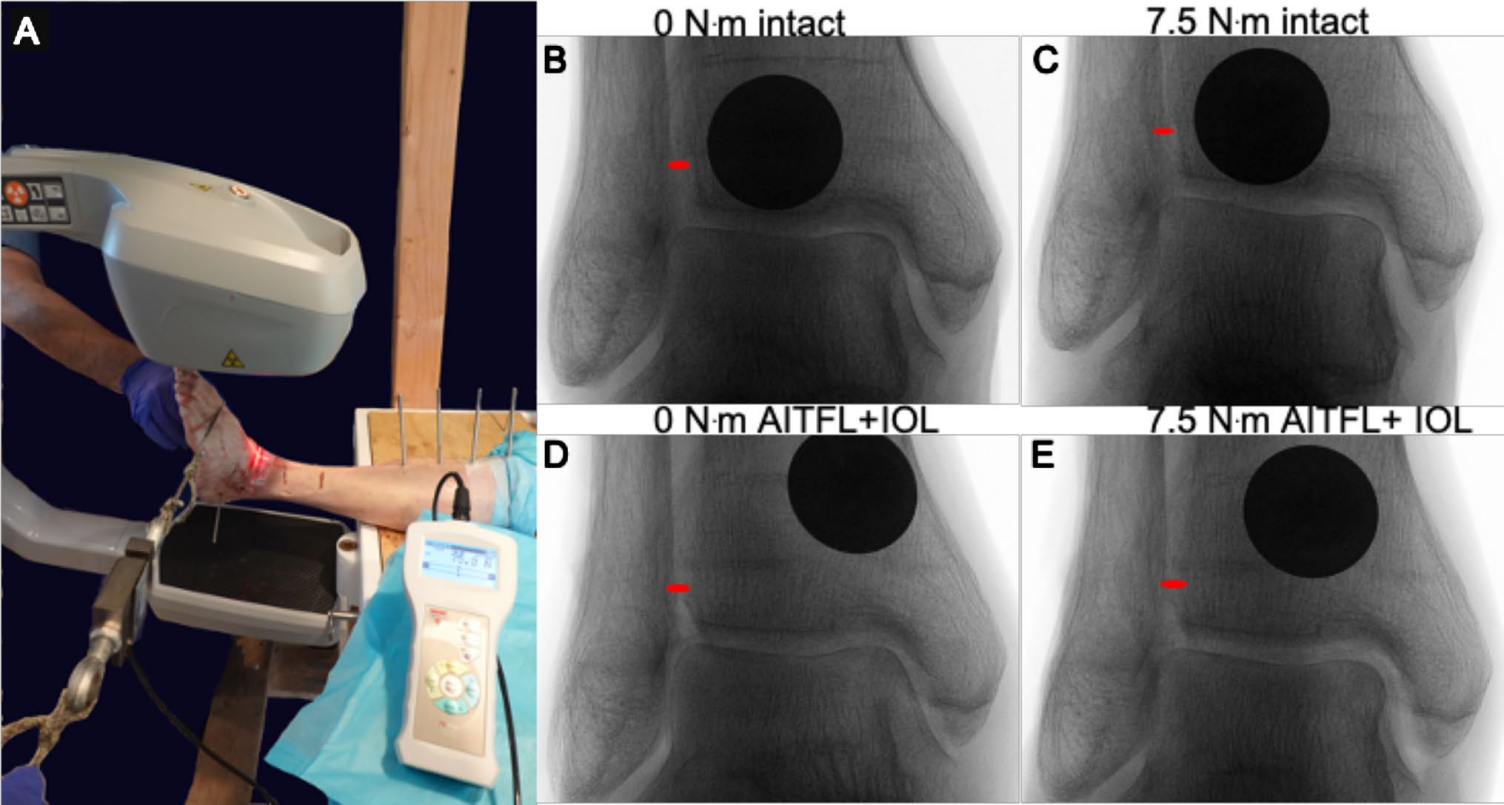
Fluoroscopic experimental setup. **A** Fluoroscopic experimental setup. **B–E** Fluoroscopic image of the TFCS at 0 and 7.5 Nm, at the intact stage (**B**, **C**) and after AITFL + IOL transection (**D**, **E**). Red pointer resembles the TFCS measurements. (Same cadaver as Fig. 1)

The TFCS was examined using a P-US probe (2D, grayscale B mode complete ultrasound; Butterfly iQ, Butterfly Network Inc, Guilford) and fluoroscopy. External rotation torque was simulated by a sharp bone hook (Arthrex, Naples, Florida) placed on the first metatarsal bone, 10 cm distal to the centre of rotation of the ankle. The centre of rotation was confirmed fluoroscopically. An external rotation directed force was then progressively applied, including 0 N (0 N^.^m), 45 N (4.5 N^.^m), 60 N (6.0 N^.^m), 75 N (7.5 N^.^m), and 90 N (9.0 N^.^m) (FB2K, Scientific Industries—Torbal Division, Oradell, NJ) (Figs. 1, 2). The foot was manually supported to hold a neutral position of the foot. It was ensured the externally directed force to be paralleled with the ground using a digital goniometer (HALO, halo medical devices HQ, Sydney, Australia). A range of external rotation torque moments was performed because the applied external rotation force in prior studies demonstrated enormous variation, and no previous study assessed the tibiofibular clear space using P-US [4, 6, 21, 38, 43]. Even though a selection of the studies used a larger external torque (up to 20 N^.^m), the maximum amount in this study did not go beyond 9 Nm because patients were not expected to tolerate more torque in the clinical setting, especially in the setting of an acute injury. The experiment and the TFCS measurements were performed by three foot and ankle specialised orthopaedic surgeons (JS, RB, and GS) and one orthopaedic resident (NH).

### Portable ultrasound

Standardised ultrasound probe and hand positions were used as previously reported [18]. The probe position was marked 10 mm above the tibiotalar joint line and positioned 30° from this transverse line with the centre covering the tibiofibular clear space. The surgical wound was not closed before application of external loads, however, the skin and soft tissue was retracted to cover the surgical site during the ultrasound experiment. Therefore, the wound would not interfere the ultrasound probe placement. Ultrasound gel was used throughout the experiment to ensure good skin contact. All dynamic ultrasound stress images at each stage of ligament transection were captured while consecutively increasing the external rotation force. After the experiment, the TFCS distances were measured from the captured images using ImageJ (National Institutes of Health). The TFCS values of each stage of ligament transection during each stressed condition were used for analysis. TFCS was determined by measuring the shortest distance between the tibia and fibula at the anterior aspect of the tibiofibular joint space (Fig. 1) [18].

### Fluoroscopy

Using fluoroscopy, true anterior to posterior views were obtained for each ligamentous transection stage at each stress moment. The TFCS distance was obtained from each radiograph using ImageJ (Fig. 2).

### Outcome measurements

After obtaining all TFCS values using both P-US and fluoroscopy images, TFCS-opening values were calculated by subtracting the intact TFCS values, unstressed and stressed, from the TFCS values of the transection stages. The TFCS-opening, therefore, shows the dynamic change after ligament transection and external rotation torque as opposed to the unstressed or stressed intact stage.

### Statistical analysis

TFCS values are presented with means and standard deviations (SD) in millimetres. In the graphs, TFCS values are presented with means and 95% confidence intervals (CI). The normality of the data was checked visually.

The TFCS values of the intact state are considered baseline values. Statistical differences between the transection stages were calculated using one-way analysis of variance (ANOVA). The post hoc Holm–Bonferroni method was used to detect which stages of ligament transection significantly differed from the intact joint. A Pearson correlation was calculated to evaluate a correlation between P-US and fluoroscopy. Interpretation to indicate the strength of correlation was considered as followed: slight correlation (*r* < 0.2), low correlation (*r* = 0.3–0.4), moderate correlation (*r* = 0.4–0.7), high correlation (*r* = 0.7–0.9), and very high correlation (*r* = 0.9–1.0) [15]. An adjusted 2-sided *p* value < 0.05 was considered statistically significant.

Two orthopaedic fellowship-trained foot and ankle surgeons performed all P-US and fluoroscopic measurements in three randomly selected specimens independently to assess interobserver agreement. To asses intraobserver reliability, one observer performed all P-US and fluoroscopic measurements in 3 randomly selected specimens twice with 12 months in between. The observers were not blinded for ligament resection stage. The inter- and intraobserver reliabilities were calculated using the intraclass correlation coefficient (ICC) through a 2-way mixed-effects model [10]. Interpretation of the ICC values was carried out according to the guidelines proposed by Shrout as follows: 0.00–0.10, virtually none; 0.11–0.40, slight; 0.41–0.60, fair; 0.61–0.80, moderate; and 0.81–1.00, substantial. The standard error of measurement (SEM) was calculated as the square root of the between-observer as well as the square root within observer variance (i.e. sum of the between-measures variance and the residual variance) [9]. In addition, the smallest detectable difference (SDD) (between observers), as well as the smallest detectable change (SDC) (within observer) was calculated from the SEM at individual level (1.96*H2*SEM).

A power analysis was conducted based on the hypothesis that a difference of minimally 2 mm in TFCS-opening would be clinically relevant, as there was no previous data available on external rotation stress assessed by P-US. To detect a true difference of 2 mm ± 2 between the paired measurements while handling a chance of having a type error of 0.05 and a type 2 error of 0.80, a sample size of 10 specimens would be required.

## Results

### P-US TFCS and TFCS-opening values

P-US TFCS values for the intact and sequential ligament sectioning stages among four torque-loading conditions are presented in Table 1. When assessed with P-US, partial syndesmotic injury encompassing the AITFL and IOL resulted in significant TFCS widening as compared to the alike stressed intact state at 4.5 Nm of external rotation torque with an average TFCS-opening of 2.6 mm ± 2.0 mm, adjusted *p* value = 0.01. With complete syndesmotic injury encompassing the AITFL, IOL, and PITFL, the TFCS widened significantly not only with an applied rotation stress, but also in the unstressed state.

**Table 1:** Ultrasonographic tibiofibular clear space values in sequential ligament sectioning stages among four torque-loading conditions

	TFCS values (mean ± SD) in mm				
Stage	0 Nm	4.5 Nm	6.0 Nm	7.5 Nm	9.0 Nm
(0) Intact	4.6 ± 1.1	5.3 ± 1.5	5.5 ± 1.0	5.7 ± 0.9	5.9 ± 0.9
(1) AITFL	5.1 ± 1.3	5.9 ± 1.3	6.2 ± 1.4	6.5 ± 1.4	7.2 ± 1.6
(2) IOL	6.1 ± 1.4	7.9 ± 2.0^a^	9.1 ± 2.5^a^	9.7 ± 2.5^a^	10.6 ± 2.5^a^
(3) PITFL	6.9 ± 1.2^a^	9.2 ± 2.3^a^	9.9 ± 2.4^a^	10.7 ± 5.0^a^	11.1 ± 2.5^a^
*p* value	0.001	< 0.001	< 0.001	< 0.001	< 0.001

*TFCS* tibiofibular clear space

^a^Corrected *p* values as compared to the alike (un-)stressed intact state are consecutively

IOL stage: *p* = 0.01, *p* = 0.001, *p* < 0.001, *p* < 0.001

PITFL stage: *p* = 0.01, *p* < 0.001, *p* < 0.001, *p* < 0.001, *p* < 0.001

### Fluoroscopy TFCS and TFCS-opening values

P-US TFCS values for the intact and sequential ligament sectioning stages among four torque-loading conditions are presented in Table 2. When assessed with fluoroscopy none of the TFCS values differed from the alike stressed intact TFCS value, Table 2.

**Table 2:** Fluoroscopic tibiofibular clear space values in sequential ligament sectioning stages among four torque-loading conditions

	TFCS values (mean ± SD) in mm				
Stage	0 Nm	4.5 Nm	6.0 Nm	7.5 Nm	9.0 Nm
Intact	4.0 ± 1.3	4.1 ± 1.3	4.2 ± 1.3	4.3 ± 1.3	4.6 ± 1.3
AITFL	3.9 ± 1.3	4.2 ± 1.2	4.5 ± 1.2	4.7 ± 1.3	5.3 ± 1.6
IOL	4.4 ± 1.1	4.9 ± 1.3	5.3 ± 1.5	5.4 ± 1.5	5.8 ± 1.5
PITFL	4.7 ± 1.5	5.4 ± 1.4	5.5 ± 1.5	5.9 ± 1.6	6.1 ± 1.7
*p* value	n.s	n.s	n.s	n.s	n.s

### Correlation between P-US and fluoroscopy

A Pearson correlation test showed a moderate correlation between P-US and fluoroscopy TFCS values with a rho of 0.52.

### Reliability

For P-US, an individual interobserver agreement of 0.95 [95% CI 0.92–0.97] for the TFCS-opening measurement was found, a SEM of 0.9, and a SDD of 2.4. The individual intraobserver agreement for P-US was 0.96 [95% CI 0.94–0.98], with a SEM of 0.7 and a SDC of 1.9.

For fluoroscopy, an individual interobserver agreement of 0.46 [95% CI 0.21–0.75] for the TFCS-opening measurement was found, a SEM of 0.9 and a SDC of 2.5. The individual intraobserver agreement for fluoroscopy was 0.56 [95% CI 0.36–0.71], with a SEM of 0.7 and a SDC of 2.0.

## Discussion

The most important findings of the present study were that P-US appears to be a useful tool in evaluating suspected syndesmotic instability, detecting widening at the distal tibiofibular articulation after both partial syndesmotic injury to the AITFL and IOL as well as complete syndesmotic disruption of the AITFL, IOL, and PITFL. Furthermore, while complete syndesmotic injuries demonstrate widening at the distal tibiofibular articulation without any applied stress, an external rotation torque of 4.5 Nm seems sufficient to detect syndesmotic instability after a partial tear involving only the AITFL and IOL.

Prior arthroscopic studies have highlighted that syndesmotic instability requires completely syndesmotic disruption of the AITFL, IOL, and PITFL, but that partial injuries to the syndesmosis (AITFL and IOL) can be rendered unstable with deltoid involvement [25, 29]. Isolated injuries to the AITFL and IOL, however, remain stable. These studies, however, relied on coronal and sagittal plane stress manoeuvres applied to the fibula rather than an external rotation torque due to the inherent challenge of externally rotating the ankle with an arthroscope in place. This study highlights the capability of P-US towards evaluating rotational instabilities of the distal tibiofibular articulation.

Subtle syndesmotic injury can cause clinically relevant instability of the ankle joint that can be associated with long-term disability and osteoarthritis when left untreated [11, 25, 29, 33, 35]. Despite the morbidity associated with syndesmotic instability, it remains challenging to identify subtle cases of instability on a large scale due to the limited accuracy, availability, or invasiveness of the assessment method [6, 17, 23]. As a consequence, no consensus has yet been reached on the definition of clinically consequential syndesmotic instability. Ultrasonography is increasingly used in the diagnosis of syndesmotic instability as it allows for a bilateral dynamic evaluation of the ankle joint at the point of care with little risk to the patient and at low cost [1, 13, 18, 32].

Three clinical studies have assessed the TFCS-opening while providing an external rotation torque to the ankle using ultrasound in patients with a complete AITFL rupture [1, 31, 40]. Baltes et al. found a mean TFCS-opening of 0.4 mm when comparing it to the unstressed injured condition and a mean TFCS-opening of 1.9 mm when comparing it to the stressed uninjured contralateral side. Their result emphasises the importance of the ability to use a contralateral side as an internal control [1]. Mei-Dan et al. and van Niekerk et al. found a TFCS-opening of 1 mm in patients with AITFL rupture [31, 40].

It is worth noting that among the advantages of P-US is its ability to evaluate the TFCS under stress. Both Baltes et al. and Mei-Dan et al. found dynamic ultrasound assessment technique to be slightly less accurate in detecting AITFL rupture as compared to the MRI, but this explicitly focuses on injury, not instability. It is the latter that drives the decision for surgical management. In this cadaveric study, no statistical difference was found between the intact and AITFL transection stage, underscoring that isolated injuries to the AITFL, whether seen on P-US or MRI, are not inherently unstable.

The TFCS-opening values detected in this study using P-US are similar to those found by Xenos et al. and Shoji et al., who also evaluated the tibiofibular diastasis after sequential transection of the syndesmosis [36, 43]. The study by Xenos et al. evaluated TFCS-opening under an external rotation torque of 5 Nm but using a Storz calliper instead of ultrasound and detected a tibiofibular opening of 1.3 (AITFL), 3.5, (IOL), and 6.3 mm after the PITFL cut as opposed to the unloaded, intact stage [43]. Thereby, shoji et al. already found a significant widening of the tibiofibular distance after the AITFL cut with external rotation stress using ultrasound [36]. The correlation between ultrasound studies and direct measurement techniques highlight the opportunity to evaluate the syndesmosis noninvasively at the point of care.

Various amounts of torque forces have been used in the literature, ranging from 0 to 20 Nm torque [4, 6, 21, 38, 43]. Given that pain will be a limiting factor in vivo when performing stress manoeuvres on an injured ankle, it is critical to be able to diagnose instability with sufficient sensitivity under the least amount of requisite force. In this study, the TFCS was measured during unloaded and after four torque-loading conditions, including 4.5, 6.0, 7.5, and 9.0 Nm. When applying 4.5 Nm, a significant increase in TFCS-opening could be detected with partial syndesmotic injury to the AITFL and IOL as compared to the intact stage. Thus, applying 4.5 Nm of external rotation force to both the injured and uninjured ankle would suffice under clinical conditions and may be better tolerated by patients than higher torque values. In contrast, the fluoroscopic results found in this study corroborate previous literature suggesting that it is insufficiently sensitive for diagnosing rotational plane syndesmotic instability [8, 23, 34].

Traditionally, the distinction between stable and unstable syndesmosis is primarily based on (1) ligament disruption as well as on (2) statistical differences in fibular translation or rotation as compared to the intact state [3, 12, 25–28, 39, 42]. New techniques such as 3D-WBCT scan, CT-scan with rotatory platforms, and (portable) dynamic ultrasonography carry the potential for reliably evaluating syndesmotic instability noninvasively [5–7, 13, 18]. The dynamic assessment method presented in this study should be considered when there is suspicion for syndesmotic instability, especially when subtle.

This study has several limitations. First, no information on the premedical history of the cadavers was available. However, no degenerative changes or injury of the ankle joint was detected using arthroscopy and fluoroscopy. Second, this study solely evaluated TFCS values after rotational torque. Syndesmotic instability is a multidirectional pathology, and other stress manoeuvres such as fibular shuck in the sagittal plane may also play a role. Third, it is unclear the degree to which patients will tolerate a 4.5 Nm external rotation torque in vivo, especially after an acute injury. Clinical studies are necessary to hone the application of P-US at the bedside. Lastly, biomechanical properties of the ankle soft tissue structures may have altered due to the freeze/thaw cycle, as well as the repetitive force loading during the experiment, which may have affected measurements obtained in this setting.

Extrapolated to the clinical setting, P-US may be a useful tool in diagnosing syndesmotic instability during external rotation stress examination clinically. 4.5 Nm of force can be used when comparing to the stressed, uninjured side, and may be better tolerated by patients than higher torque values. The clinical instability cutoff values should be further investigated in a clinical research setting.

## Conclusion

P-US is a useful tool in diagnosing syndesmotic instability during external rotation stress examination. TFCS-opening increased as additional ligaments of the syndesmosis were transected, and application of 4.5 Nm torque was sufficient to detect a difference of 2.6 mm after the IOL cut.

## Conflict of interest

The authors disclose non-financial research support from Butterfly Network Inc.

## Ethical approval

The use of cadaveric tissue models for biomechanical testing was exempt by the IRB 2016P001295/MGH.

## Informed consent

Not applicable.

## Abbreviations

AITFL: Anterior interior tibiofibular ligament
ANOVA: One-way analysis of variance
CI: Confidence intervals
ICC: Intraclass correlation coefficient
IOL: Interosseous ligament
TFCS: Tibiofibular clear space
PITFL: Posterior–interior tibiofibular ligament
P to A: Posterior to anterior
P-US: Portable ultrasonography
WBCT: Weight-bearing computed tomography
3D: Three-dimensional
